# Supplementary material for: Creating synthetic spaces for higher-order topological sound transport
Source: Nat Commun. 2021 Aug 19;12:5028. doi: 10.1038/s41467-021-25305-z (PMC8377151; doi:10.1038/s41467-021-25305-z)
Supplement: Supplementary file 1 — Supplementary Information [file 41467_2021_25305_MOESM1_ESM.pdf]

Supplementary Information for
Creating synthetic spaces for higher-order topological sound transport

Hui Chen[1], Hongkuan Zhang[2], Qian Wu[1], Yu Huang[2], Huy Nguyen[1],
Emil Prodan[3*], Xiaoming Zhou[2*], Guoliang Huang[1*]
[1]Department of Mechanical and Aerospace Engineering, University of Missouri, Columbia, MO 65211, USA
[2]School of Aerospace Engineering, Beijing Institute of Technology, Beijing 100081, China
[3]Department of Physics, Yeshiva University, New York, NY 10016, USA
These authors contributed equally: Hui Chen, Hongkuan Zhang
*Corresponding authors: prodan@yu.edu (E.P.); zhxming@bit.edu.cn (X.Z.); huangg@missouri.edu (G.H.)

Contents

Supplementary Note1. Tight-binding model and Chern number 2
A. Reduction to 1D tight-binding models 2
B. 1D topological pumping and first Chern number 3
C. 2D topological pumping and second Chern number 5
D. Modulated channels with loss 7

Supplementary Note2. Discussion on adiabaticity 8

Supplementary Note3. Directional and path-dependent topological pumping 10

Supplementary Note4. Robustness to disorder in pumping parameters 11

References 15

Supplementary Note1. TIGHT-BINDING MODEL AND CHERN NUMBER

In this section, we supply the details of the reduction procedure, review Chern numbers related to the quantum Hall effect and derive the phase diagrams of the 1D and 2D topological pumping lattices within the nearest-neighbour tight-binding (TB) model. For a 1D topological system with one pumping parameter in a synthetic dimension, one can mathematically map the 1D pumping to the 2D quantum Hall effect with a first Chern number. Similar to the 1D case, a 2D topological pumping can be subject to two pumping parameters, corresponding to a 4D quantum Hall effect, which is characterized by a second Chern number.

A. Reduction to 1D tight-binding models

The basic acoustic cavity used in our designs, the closed cube, displays a small number of acoustic resonant modes Q_α with frequency ν_α in the frequency range from 0 to 10 kHz. A tight-binding representation of the acoustic wave propagation across our meta-crystal can be achieved via the mode coupling technique. In this approach, the pressure distribution of a collective resonant mode at frequency ω is expanded as

$$\Psi(x,z;\omega) = \sum_{n,m,\alpha} \psi^\alpha_{nm}\, Q^{nm}_\alpha(x,z), \tag{S1}$$

where Q^{nm}_α is the resonant mode α supported by the acoustic cavity located at address (n,m) in our 2D structure (analysis of the 3D structure proceeds similarly). The complex coefficient ψ^α_{nm} encodes the amplitude and the phase of that resonant mode. Same expansion can written in a more abstract notation as

$$|\Psi\rangle = \sum_{n,m,\alpha} \psi^\alpha_{nm} |n,m,\alpha\rangle. \tag{S2}$$

Then the dynamical matrix of the collective resonant modes takes the generic form $D = D_x + D_z$, with

$$D_x = \sum_{n,m,\alpha,\beta} \kappa^x_{\alpha\beta}(h_{nm})\big(|n,m,\alpha\rangle\langle n+1,m,\beta| + |n+1,m,\alpha\rangle\langle n,m,\beta|\big) \tag{S3}$$

and

$$D_z = \sum_{n,m,\alpha} \nu^2_\alpha |n,m,\alpha\rangle\langle n,m,\alpha| + \sum_{n,m,\alpha,\beta} \kappa^z_{\alpha,\beta}\big(|n,m,\alpha\rangle\langle n,m+1,\beta| + |n,m+1,\alpha\rangle\langle n,m,\beta|\big). \tag{S4}$$

Note that the coupling coefficients are symmetric in the α and β indices. We recall that the horizontal coupling coefficients depend on the thickness h_{nm} of the connecting channel between (n,m) and $(n+1,m)$ cavities, which is modulated in both x and z directions. On the other hand, the coupling coefficients in the z-direction are uniform.

Because of the z-variation of the horizontal coupling coefficients, the dynamical matrix does not commute with the z-translations, hence Bloch decomposition is not immediately available. We recall that the horizontal connecting channels are modulated with the algorithm

$$h_{nm} = h_0[1 + \delta \sin(b_{n \bmod 3} + \phi_m)], \tag{S5}$$

hence the z-variation comes from the dependence on the pumping parameter ϕ. We can always seek the eigen-modes of the dynamical matrix in the form $|\Psi\rangle = \int dk_z\, |\Psi(k_z)\rangle$, with

$$|\Psi(k_z)\rangle = \sum_{n,m,\alpha} e^{\imath k_z m} \varphi^\alpha_{nm} |n,m,\alpha\rangle, \tag{S6}$$

and observe that

$$D_z|\Psi(k_z)\rangle = \sum_{n,m,\alpha,\beta} \Big[\delta_{\alpha\beta}\nu^2_\alpha e^{\imath k_z m}\varphi^\beta_{nm} + \kappa^z_{\alpha\beta}\Big(e^{\imath k_z(m+1)}\varphi^\beta_{n(m+1)} + e^{\imath k_z(m-1)}\varphi^\beta_{n(m-1)}\Big)\Big]|n,m,\beta\rangle. \tag{S7}$$

If ϕ is sampled very finely along the z-direction as described in the main text, then

$$\varphi^\beta_{n(m\pm1)} \approx \varphi^\beta_{nm} \pm \epsilon a_z \phi'\, \partial_\phi \varphi^\beta_{nm} \tag{S8}$$

and we have

$$D_z|\Psi(k_z)\rangle = \sum_{n,m,\alpha,\beta}[\nu_\alpha^2\delta_{\alpha\beta} + 2\cos(k_z)\kappa^z_{\alpha\beta} + 2\epsilon i a_z\phi'\sin(k_z)\kappa^z_{\alpha\beta}\partial_\phi]e^{\imath k_z m}\varphi^\beta_{nm}|n,m,\beta\rangle. \tag{S9}$$

The full COMSOL simulations reported in Fig. 1c indicate that the spectrum in our window of interest can be explained by one mode per acoustic cavity and this is quantitatively demonstrated in Supplementary Figure 1. As such, we can drop the α and β indices. Then, to the zero-th order in ϵ, $D_z|\Psi(k_z)\rangle = \nu(k_z)^2|\Psi(k_z)\rangle$, with $\nu(k_z)^2 = \nu_0^2 + 2\cos(k_z)\kappa_0^z$ and the action of the full dynamical matrix becomes

$$D\sum_{n,m}e^{\imath k_z m}\varphi_{nm}|n,m\rangle = \sum_{n,m}e^{\imath k_z m}\nu(k_z)^2\varphi_{nm}|n,m\rangle + \sum_{n,m}e^{\imath k_z m}\kappa^x(h_{nm})[\varphi_{(n+1)m}|n,m\rangle + \varphi_{nm}|n+1,m\rangle]. \tag{S10}$$

The resonant condition $D|\Psi\rangle = f^2|\Psi\rangle$ is satisfied if the φ-coefficients are solutions of the equation

$$(f^2 - \nu(k_z)^2)\varphi_{nm} = k^x(h_{nm})\varphi_{(n+1)m} + k^x(h_{(n-1)m})\varphi_{(n-1)m}. \tag{S11}$$

Hence, for a fixed m, φ_{nm} is an eigen-mode of reduced Hamiltonian $H(\phi_m)$ written in Eq. 4 of the main text. If eigenvalue of this eigen-mode is ϵ_m, then the value of k_z at the layer m is determined by the relation $f^2 - \nu(k_z)^2 = \epsilon_m$. Let us acknowledge that, for weak modulations of h_{nm}, we have:

$$\kappa^x(h_{nm}) \approx \kappa^x(h_0) + \frac{\mathrm{d}\kappa^x}{\mathrm{d}h}h_0\delta\cos(b_{n \bmod 3} + \phi_m) = \kappa_0[1 + \delta'\cos(b_{n \bmod 3} + \phi_m)]. \tag{S12}$$

Inserting back the term in Eq. S9 that is neglected in the zero-th order analysis, we find that, in the first order in ϵ, the dispersion equation reads

$$-i\epsilon(2a_z\phi'\sin(k_z(\phi))\kappa_0^z)\partial_\phi|\varphi\rangle = \big(H(\phi) - \epsilon(\phi)\big)|\varphi\rangle, \tag{S13}$$

where all the dependencies on m are passed to the pumping parameter ϕ.

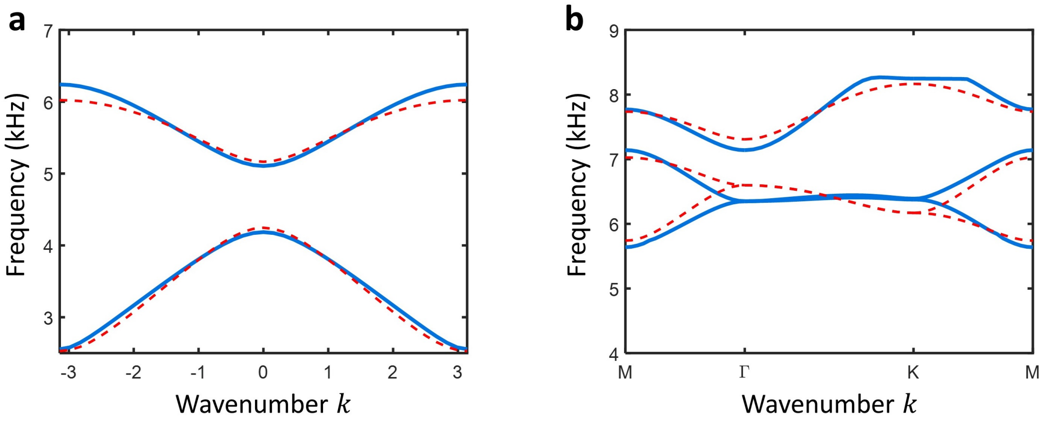

Supplementary Figure 1: **Two highest bands of 1D pumping (a) and 2D pumping (b) when the pumping parameter is $\phi = -0.2\pi$.** The blue solid lines are the full-wave simulation results. The corresponding fitting results are in red dashed lines. The fitting parameters for 1D pumping are $\varepsilon = 3.61$, $\kappa_0 = -1.44$ and $\delta = 0.55$. The fitting parameters for 2D pumping are $\varepsilon = 4.43$, $\kappa_0 = -0.91$ and $\delta = 0.55$.

B. 1D topological pumping and first Chern number

The bulk Hamiltonian of the 1D 3-periodic lattice (Supplementary Figure 2a) with a space-dependent modulation on the hopping is

$$H(\phi) = \begin{bmatrix} \varepsilon & \kappa_1 & \kappa_3 e^{-ik} \\ \kappa_1 & \varepsilon & \kappa_2 \\ \kappa_3 e^{ik} & \kappa_2 & \varepsilon \end{bmatrix}, \tag{S14}$$

where k is the wave number, ε represents the on-site potential, and $\kappa_j(\phi) = \kappa_0[1 + \delta\cos(b_j + \phi)](j = 1, 2, 3)$ are modulated hopping amplitudes with bare hopping κ_0, modulation amplitude δ, and modulation frequency b_j. The pumping parameter ϕ corresponds to an additional synthetic dimension. We then develop this TB model to achieve the associated acoustic dispersion relation. It is found that altering the thickness of channel is equivalent to changing the coupling coefficients between neighbouring sites, while the on-site energy does not change due to the fixed size of the cavity. The on-site potential ε ($\varepsilon = 3.61$) and the coupling hopping κ_0 ($\kappa_0 = -1.44$) are extracted from full-wave finite element simulations. As expected, the dispersion curves calculated from the TB model are quantitatively agree with the numerical simulations (Supplementary Figure 1a). Therefore, our coupled acoustic cavities can be faithfully described by the TB model.

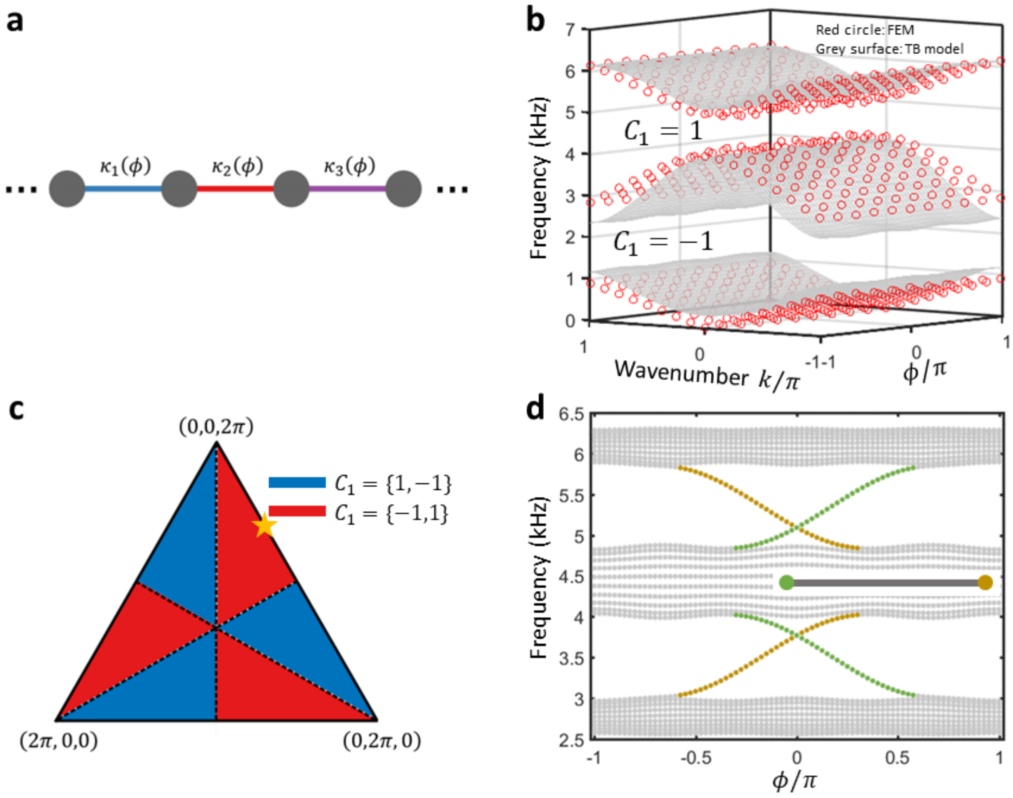

Supplementary Figure 2: **Tight-binding model and phase diagram of 1D space-modulated lattices.** **a** Schematic of the 1D topological pumping lattice with a 3-periodic unit cell, which can be modulated using the pumping parameter ϕ; and **b** its corresponding bulk band structure as a function of the nondimensional wavenumber and the pumping parameter ϕ with first gap Chern numbers assigned to each band gap. The grey surfaces are the full-wave simulation results. The corresponding fitting results of TB model are in red circles. The fitting parameters are $\varepsilon = 3.61$, $\kappa_0 = -1.44$ and $\delta = 0.55$. **c** Phase diagram of the 1D topological pumping lattice illustrating first gap Chern numbers for the lower and upper band gaps as a function of modulation frequencies $\{b_1, b_2, b_3\}$ interpreted as barycentric coordinates in the plane $(b_1 + b_2 + b_3 = 2\pi)$. The yellow star denotes the phase with the specific parameters $(\{b_1, b_2, b_3\} = \{0, 2\pi/3, 4\pi/3\})$ used in **b** and **d**. **d** The edge-state dispersion of a finite lattice. The green and yellow dots indicate left and right edge modes, respectively.

Supplementary Figure 2b shows the evolution of dispersion surfaces as a function of the nondimensional wavenumber and the pumping parameter. The coupling between the modulated dimension and the synthetic dimension opens two bulk gaps and can induce topological phase transitions in the model. The quantized bulk response of this system is characterized by the first gap Chern number: the sum of first Chern numbers of the bands below that gap in frequency [1]

$$C_1 = \sum_{f_\beta < f_{\text{gap}}} \frac{1}{2\pi i} \int_{\text{BZ}} B^{\beta}_{lm}(\mathbf{k}) \mathrm{d}^2\mathbf{k}, \tag{S15}$$

which is an integral on the 2D Brillouin zone (BZ) of the Berry curvature

$$B^{\beta}_{lm}(\mathbf{k}) = \partial_{k_l} A^{\beta}_{m}(\mathbf{k}) - \partial_{k_m} A^{\beta}_{l}(\mathbf{k}), \tag{S16}$$

where $A^{\beta}_{l} = \langle \psi^{\beta}(\mathbf{k}) | \partial_l | \psi^{\beta}(\mathbf{k}) \rangle$ is the Berry connection. Here $|\psi^{\beta}(\mathbf{k})\rangle$ enotes the eigenstate of the band β and of Bloch momentum $\mathbf{k} = (\phi, k)$. The first gap Chern number is an integer and invariant as long as the band gap does not close. The first gap Chern number manifests through the quantization of the bulk response, which has the corresponding edge pumping. Therefore, gapless and unidirectional edge states appear in a finite sample (as many as the sum of the Chern numbers of bands below a given gap). To demonstrate the robustness of the first gap Chern number, Supplementary Figure 2c shows the phase diagram of the first gap Chern number in function of pumping parameters $\{b_1, b_2, b_3\}$ interpreted as barycentric coordinates in the plane $(b_1 + b_2 + b_3 = 2\pi)$. It is noticed that only two arrays of the first gap Chern numbers, namely $\pm(1, -1)$ are obtained with different relative values of the modulation parameters b_j, $j = 1$, 2, and 3. This figure also reveals that the first gap Chern number is insensitive to uncertainty in the modulation parameters except for near critical lines where phase transitions occur. The edge-state dispersion of a finite lattice with $C_1 = \{-1, 1\}$ is plotted in Supplementary Figure 2d and a pair of edge states are populated within two bulk band gaps.

C. 2D topological pumping and second Chern number

Here, we derive the second Chern number in a 2D topological pumping lattice (Supplementary Figure 3a):

$$H(\phi) = \sum_{x,y} \varepsilon \, |x, y\rangle \langle x, y| + \sum_{x,y} [\kappa_x(\phi^x) \, |x, y\rangle \langle x + 1, y| + \kappa_y(\phi^y) \, |x, y\rangle \langle x, y + 1| + \text{h.c.}]. \tag{S17}$$

This Hamiltonian H of this system can be decomposed into two orthogonal 1D AAH model along the two modulated directions x and y

$$H = H(\phi^x) \otimes I + I \otimes H(\phi^y) = \begin{bmatrix} \varepsilon & \kappa^x_1 & \kappa^x_3 e^{-ik_x} & \kappa^y_1 & 0 & 0 & \kappa^y_1 e^{-ik_y} & 0 & 0 \\ \kappa^x_1 & \varepsilon & \kappa^x_2 & 0 & \kappa^y_1 & 0 & 0 & \kappa^y_2 e^{-ik_y} & 0 \\ \kappa^x_3 e^{ik_x} & \kappa^x_2 & \varepsilon & 0 & 0 & \kappa^y_1 & 0 & 0 & \kappa^y_3 e^{-ik_y} \\ \kappa^y_1 & 0 & 0 & \varepsilon & \kappa^x_1 & \kappa^x_3 e^{-ik_x} & \kappa^y_2 & 0 & 0 \\ 0 & \kappa^y_1 & 0 & \kappa^x_1 & \varepsilon & \kappa^x_2 & 0 & \kappa^y_2 & 0 \\ 0 & 0 & \kappa^y_1 & \kappa^x_3 e^{ik_x} & \kappa^x_2 & \varepsilon & 0 & 0 & \kappa^y_2 \\ \kappa^y_1 e^{ik_y} & 0 & 0 & \kappa^y_2 & 0 & 0 & \varepsilon & \kappa^x_1 & \kappa^x_3 e^{-ik_x} \\ 0 & \kappa^y_3 e^{ik_y} & 0 & 0 & \kappa^y_2 & 0 & \kappa^x_1 & \varepsilon & \kappa^x_2 \\ 0 & 0 & \kappa^y_3 e^{ik_y} & 0 & 0 & \kappa^y_2 & \kappa^x_3 e^{ik_x} & \kappa^x_2 & \varepsilon \end{bmatrix}, \tag{S18}$$

where $\kappa^x_j = \kappa_0[1 + \delta \cos(b^x_j + \phi^x)]$ and $\kappa^y_j = \kappa_0[1 + \delta \cos(b^y_j + \phi^y)](j = 1, 2, 3)$ are modulated hopping terms. In this TB model, each $H(\phi^{\alpha})$ is a 1D Hamiltonian with one pumping parameter, that is, 1D topological pumping. Because each 1D pumping has bulk and edge states, the eigenstates in 2D pumping can be grouped into three modes [2]: (i) 2D bulk modes are the product of 1D bulk modes in x and y directions; (ii) 1D topological edge modes are the product of 1D bulk modes with a zero corner modes; (iii) 0D topological corner modes are the product of two corner modes in 1D pumping.

We then implement this 2D TB model to achieve the associated acoustic dispersion relation. The on-site potential is fitted as $\varepsilon = 4.43$, and the hopping parameter as $\kappa_0 = -0.91$. As we can see in Supplementary Figure 1b, the TB model can capture the dispersion relations of real acoustic material very well in the whole Brillouin zone. Supplementary Figure 3b shows the band dispersion of the model along high symmetry lines (X-Γ-M-X) and synthetic dimensions $(\phi = \phi^x = \phi^y)$. The coupling between modulated dimensions and synthetic dimensions opens four bulk gaps and can be characterized with non-trivial second gap Chern numbers [2, 3]:

$$C_2 = \sum_{f_\beta < f_{\text{gap}}} \frac{1}{32\pi^2} \int_{\text{BZ}} \varepsilon_{lmno} B^{\beta}_{lm} B^{\beta}_{no}(\mathbf{k}) \mathrm{d}^4\mathbf{k}, \tag{S19}$$

where ε_{lmno} is the 4D Levi-Civita symbol, ensuring that this topological invariant vanishes in lower dimensions. Here the integral on the 4D Brillouin zone (BZ) of the Berry curvature $B^{\beta}_{lm}(\mathbf{k}) = \partial_{k_l} A^{\beta}_{m}(\mathbf{k}) - \partial_{k_m} A^{\beta}_{l}(\mathbf{k})$ is written in terms of the Berry connection $A^{\beta}_{l} = \langle \psi^{\beta}(\mathbf{k}) | \partial_l | \psi^{\beta}(\mathbf{k}) \rangle$. The Bloch momentum is $\mathbf{k} = (\phi^x, \phi^y, k_x, k_y)$. Supplementary Figure 3c shows the phase diagram of the second gap Chern number in function of the modulation frequency b^{α}_j

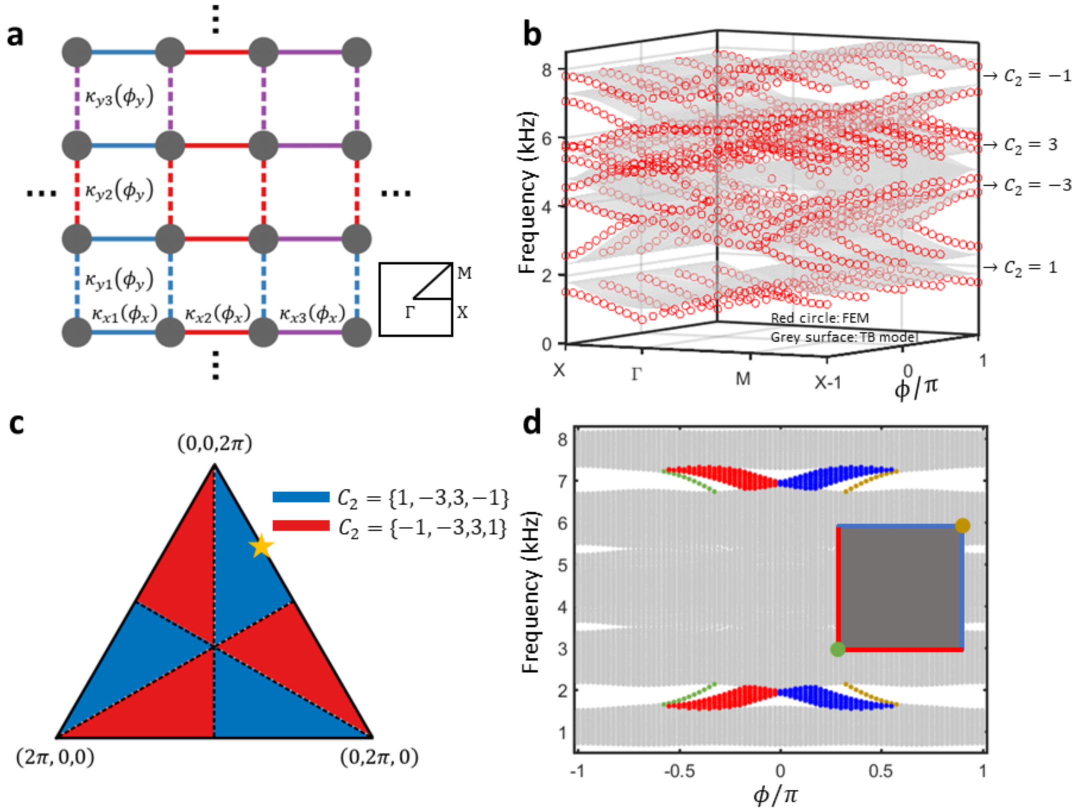

Supplementary Figure 3: **Tight-binding model and phase diagram of 2D space-modulated lattices.** **a** Schematic of the 2D topological pumping lattice with a 3×3 unit cell, which can be modulated using the pumping parameters ϕ^x and ϕ^y; and **b** its corresponding bulk band structure along high symmetry lines and the synthetic dimension ϕ with second gap Chern numbers assigned to four band gaps. The grey surfaces are the full-wave simulation results. The corresponding fitting results are in red circles. The fitting parameters are $\varepsilon = 4.43$, $\kappa_0 = -0.91$, $\delta = 0.55$. **c** Phase diagram of the 2D topological pumping lattice illustrating second gap Chern numbers for four band gaps as a function of modulation frequencies $\{b_1, b_2, b_3\}$ interpreted as barycentric coordinates in the planes $((b_1^\alpha + b_2^\alpha + b_3^\alpha = 2\pi)$ with $\alpha \in \{x, y\}$; $b_j^x = b_j^y$, $j = 1,\ 2$, and 3). The yellow star denotes the phase with the specific parameters $(\{b_1^\alpha, b_2^\alpha, b_3^\alpha\} = \{0, 2\pi/3, 4\pi/3\})$ used in **b** and **d**. **d** The edge- and corner-state dispersions of a finite lattice. The green and yellow dots indicate left-bottom and right-top corner modes, respectively. The colors of modes indicate their characteristics, which are represented in the inset.

interpreted as barycentric coordinates. It is found that only two arrays of the second gap Chern numbers for four band gaps, namely $\pm(1, -3, 3, -1)$ are obtained with different relative values of the modulation parameters. Thus, one can tune the system across a topological transition point by changing the value of the modulation parameter. The edge- and corner-state dispersion of a finite lattice is shown in Supplementary Figure 3d. Indeed, the effective Hamiltonian of Eq. S18 implies that the corner mode are composed of the product of two edge modes in the 2D system [2]. As a result, the topological corner modes in the 3D system are directly related to the nonzero combinations of two first gap Chern numbers, $C \equiv (C_1^x, C_1^y)$, which are further connected to the second gap Chern number. It reads [4]

$$\sum_{i=1}^{r} C_{2,f_i} = \sum_{f_{r-1}<f_x+f_y<f_r} C_{1,f_x}^x C_{1,f_y}^y, \tag{S20}$$

where C_{2,f_i} is the second Chern number for the band gap with the frequency no larger than f_i, and C_{1,f_x}^x is the first Chern number for the band gap with the frequency no larger than f_x. In our system, the topological invariants protecting the corner modes from the lowest to the highest band gaps are $(C_{1,f_1}^x, C_{1,f_1}^y) = (-1,-1)$, $(C_{1,f_1}^x, C_{1,f_2}^y) = (-1,1)$, $(C_{1,f_2}^x, C_{1,f_1}^y) = (1,-1)$, and $(C_{1,f_2}^x, C_{1,f_2}^y) = (1,1)$.

D. Modulated channels with loss

We evaluate the loss effects on topological boundary modes by introducing loss in the hopping $\kappa_j(\phi) = \kappa_0[1 + \delta\cos(b_j + \phi)] + i\eta$, where η is the loss coefficient. Supplementary Figure 4 shows the edge-state dispersion of the 2D system with different loss coefficients. In Supplementary Figure 4a, it is clearly evident that the smaller loss coefficient with $\eta = 0.1\kappa_0$ cannot affect the dispersion property by comparing with the lossless case in Supplementary Figure 2d. However, as seen in Supplementary Figure 4b, the larger loss seriously affected the dispersion property, which makes the boundary mode and bulk mode overlap. This phenomenon is further confirmed in the 3D system; see Supplementary Figure 5.

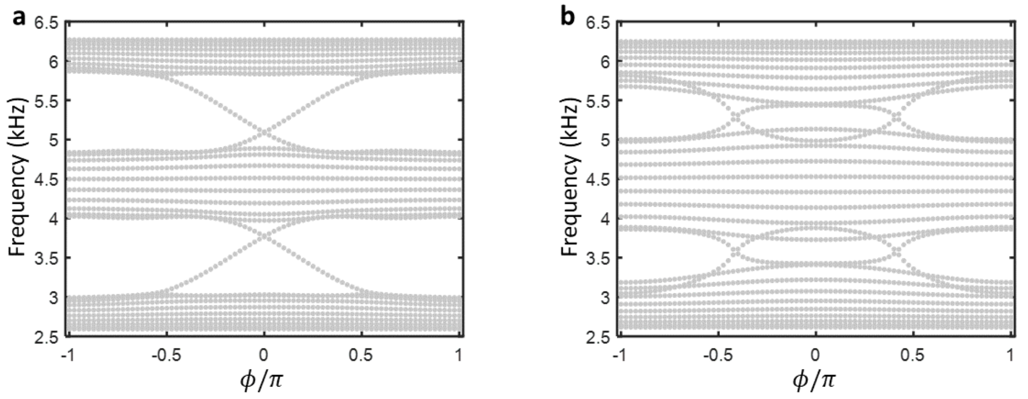

Supplementary Figure 4: **The edge-state dispersion of the 2D channel-modulated acoustic crystal with different losses in the hopping. a** $\eta = \kappa_0$. **b** $\eta = 6\kappa_0$.

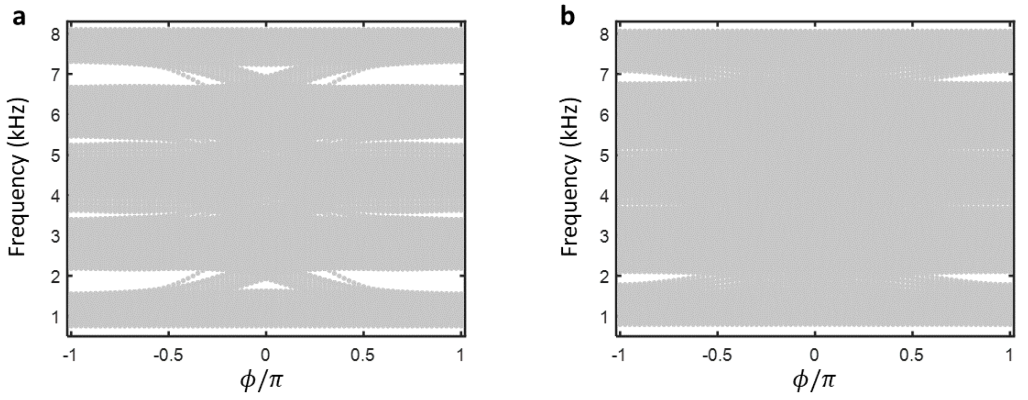

Supplementary Figure 5: **The edge- and corner-state dispersion of the 3D channel-modulated acoustic crystal with different losses in the hopping. a** $\eta = \kappa_0$. **b** $\eta = 3\kappa_0$.

Supplementary Note2. DISCUSSION ON ADIABATICITY

In order to assess the adiabatic regime, we calculate the ratio of the pressure amplitude between input A and output B with different numbers of horizontal layers; see Supplementary Figure 6a. It is observed that for topological pumping the energy is reliably transported from the left edge to the right edge when $L_z > 10a_z$; see Supplementary Figure 6b. In contrast, when $L_z < 10a_z$, the amplitudes of the waveguide drop. Then the adiabatic condition may fail and the reflection between different layers will affect the topological waveguide. Further, we present simulation results of the edge-to-edge and corner-to-corner pumping in Figs. 7 and 8, respectively. It is also observed that our 3D system with $L_z = 15a_z$ has a reliable sound transport.

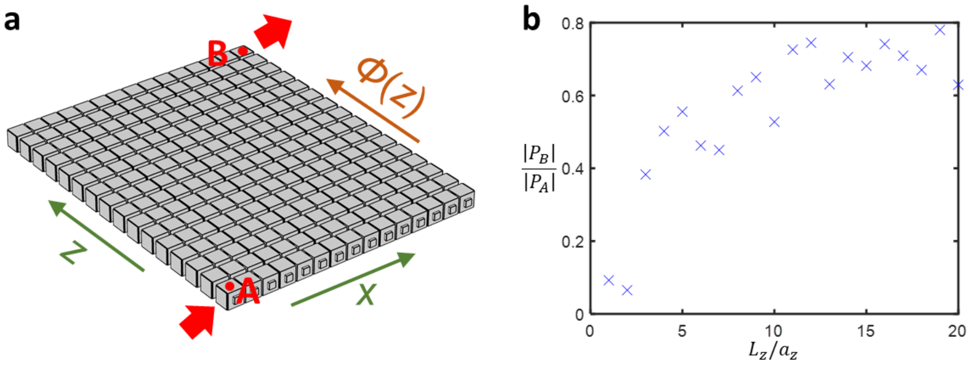

Supplementary Figure 6: **Assessing adiabaticity of the 1D topological pumping.** **a** Schematic of the 2D channel-modulated acoustic crystal with input A and output B. **b** Ratio of the pressure amplitude between A and B with different numbers of horizontal layers. The pressure is imposed at A and collected at B.

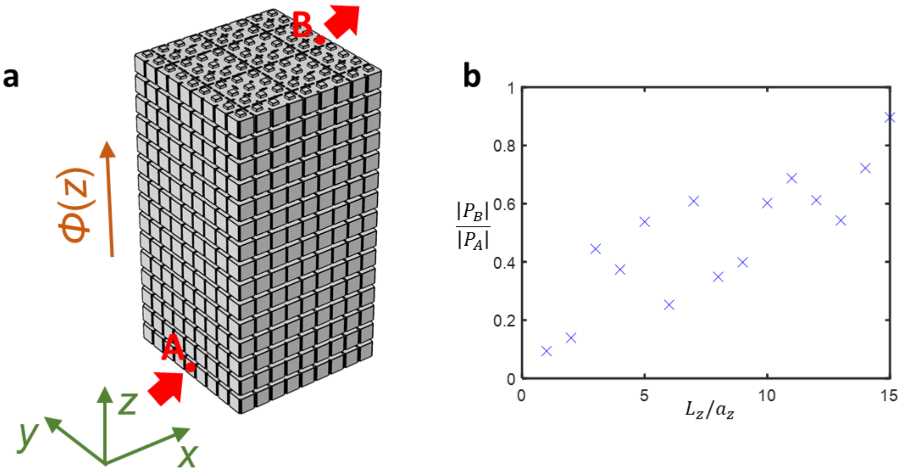

Supplementary Figure 7: **Assessing adiabaticity of the edge-to-edge topological pumping.** **a** Schematic of the 3D channel-modulated acoustic crystal with input A and output B. **b** Ratio of the pressure amplitude between A and B with different numbers of horizontal layers. The pressure is imposed at A and collected at B.

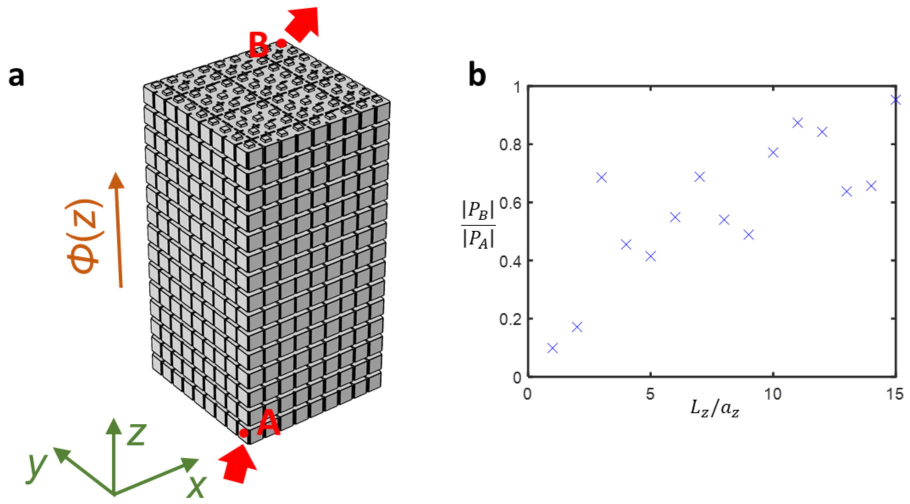

Supplementary Figure 8: **Assessing adiabaticity of the corner-to-corner topological pumping.** **a** Schematic of the 3D channel-modulated acoustic crystal with input A and output B. **b** Ratio of the pressure amplitude between A and B with different numbers of horizontal layers. The pressure is imposed at A and collected at B.

Supplementary Note3. DIRECTIONAL AND PATH-DEPENDENT TOPOLOGICAL PUMPING

The phason plays the role of synthetic momenta and this implies that the pumping process along a given orbit manifests only in a particular space direction. To demonstrate that the pumping processes are indeed highly directional, we numerically investigate the acoustic characteristics of the 3D structure for different phason orbits and with the source placed at different space locations. If the pumping parameters are held constant with $\phi^x = \phi^y = -0.2\pi$ from the bottom array to the top array along the vertical direction (Supplementary Figure 9a), we excite the acoustic waves at two different edges with the frequency at $f = 7498$ Hz: one at the centre of the left-bottom edge (Supplementary Figure 9b) and the other at the centre of the right-bottom edge (Supplementary Figure 9c). The resulting acoustic pressure distributions show the sound stays confined to the same side and it cannot spread to the bulk. When we excite the acoustic waves at the bottom corner with the frequency at $f = 6175$ Hz, the sound stays confined to the corner and is transferred to the top corner along the edge (Supplementary Figure 9d). We then present an additional 3D acoustic structure, engineered to pump along the horizontal orbit from $(-0.2\pi, -0.2\pi)$ to $(0.2\pi, -0.2\pi)$ (Supplementary Figure 9e). We note that when the sound is excited at the left-bottom edge, it can be pumped to the bulk, and then to the opposite top-right edge (Supplementary Figure 9f). However, when the sound is excited at the right-bottom edge, it still stays confined to the same side because ϕ_y is not pumped (Supplementary Figure 9g). In addition, we observe that when the sound is excited at the bottom corner, it is propagated along the same surface instead to the opposite corner at the top layer (Supplementary Figure 9h). Similar observations hold if the orbit is changed from $(-0.2\pi, -0.2\pi)$ to $(-0.2\pi, 0.2\pi)$.

To further demonstrate the directional character of the pumping, we vary the pumping parameters along the diagonal orbit (see the inset of Fig. 2d in the main text). As shown in Supplementary Figure 10a (or Fig. 3b), the edge-to-edge topological pumping is observed with the source placed on the left-bottom edge at $f = 7498$ Hz, while it is not allowed to pump when the source is moved to the right-bottom edge. Similar results can be observed from Supplementary Figure 10c (or Fig. 3d) for the 2D topological pumping on the propagation of corner states from the left-bottom corner to the right-top corner with the frequency at $f = 6175$ Hz. However, acoustic wave is heavily suppressed if the the source is placed the right-bottom corner (Supplementary Figure 10d). In this respect, the structure acts like a perfect "transistor" because the pumping can be completely turned on and off by 180° rotations. Similar observations hold if the orbit is aligned with the second diagonal $\phi^x = -\phi^y$.

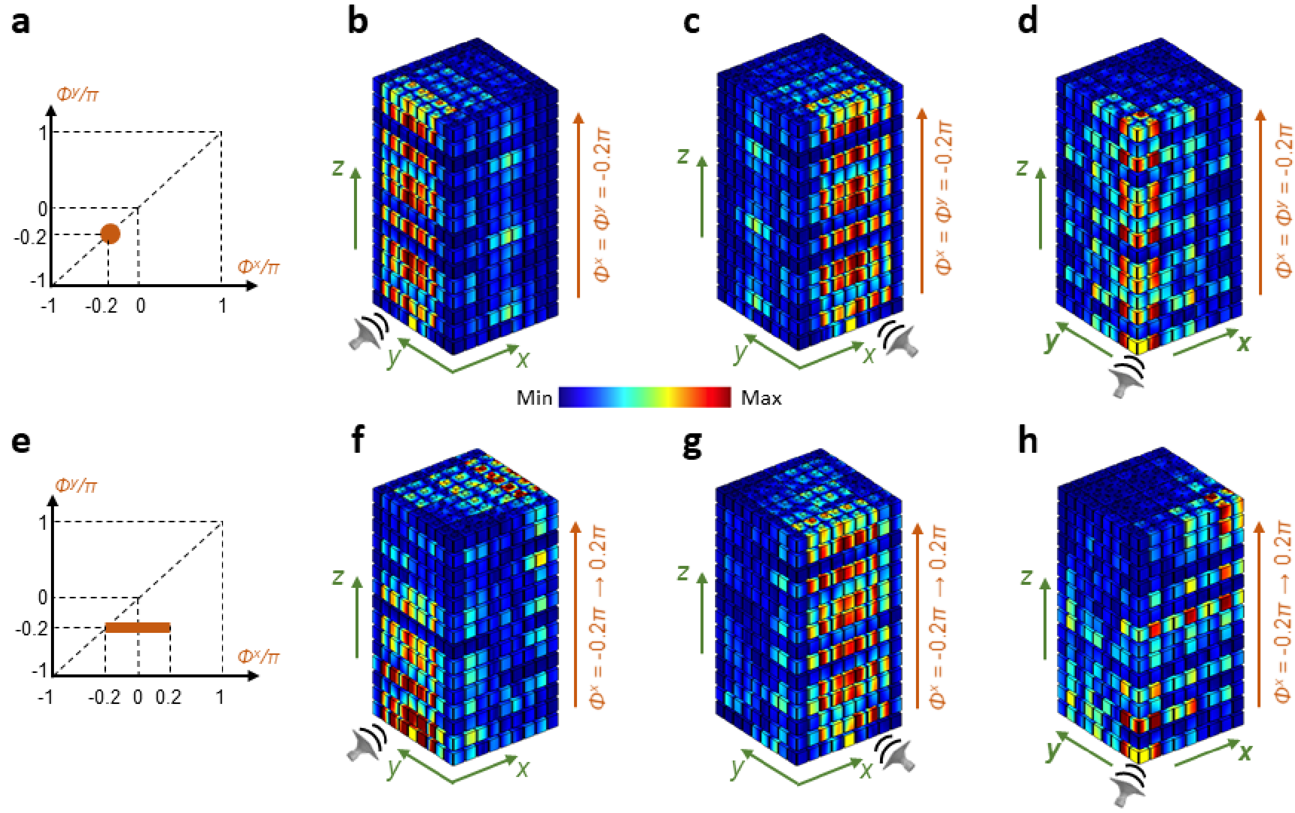

Supplementary Figure 9: **Numerical observation of edge-to-edge topological and corner-to-corner topological pumpings for different phason orbits and with the source placed at different space locations.** **a** A phason orbit with $\phi_x = \phi_y = -0.2\pi$ and its corresponding 3D acoustic structure (b-d). Sound that is excited at the left-bottom edge **b** or the right-bottom edge **c** with the frequency at $f = 7498$ Hz excites the topological edge modes and spreads out along the facet. **d** Sound that is excited at the bottom corner with the frequency at $f = 6175$ Hz excites the topological corner modes and spreads out along the edge. **e** A horizontal phason orbit (from $(-0.2\pi, -0.2\pi)$ to $(0.2\pi, -0.2\pi)$) and its corresponding 3D acoustic structure (f-h). **f** Sound is excited at the left-bottom edge to be pumped to the right-top edge with the frequency at $f = 7498$ Hz, while **g** no such pumping is observed when sound is excited at the right-bottom edge. **h** Sound is excited at the bottom corner to be pumped to the opposite top corner along the x direction with the frequency at $f = 6175$ Hz.

Supplementary Note4. ROBUSTNESS TO DISORDER IN PUMPING PARAMETERS

In this section, we discuss the robustness of topologically protected pumping against random disorders of pumping parameters. We allow pumping parameters of the m-th layer to be randomly varied $\delta\phi_m$ with respect to its original values (ϕ_m, ϕ_m); that is, $\phi_m^x = \phi_m^y = \phi_m + \delta\phi_m$. The disorder degree is defined as $100\% \max(\delta\phi_m/\phi_m)$. In the simulation of disordered acoustic pumping, the disorder parameters are generated by the random function in MATLAB script and converted to small deviations of pumping parameters with 10% disorder. Here, the perturbed pumping parameters are $\delta\phi_m = \{-0.008, -0.0012, -0.0108, 0.0138, -0.0122, -0.0110, -0.0132, -0.0109, -0.0026, -0.0076, 0.0169, -0.0028,$ $-0.0126, 0.0162, 0.0192\}\pi$ and ϕ_m varies linearly from -0.2π to 0.2π along the z direction. It is seen that the 10% disorder degree has no noticeable influence on the edge-to-edge (Supplementary Figure 11a) and corner-to-corner (Supplementary Figure 11b) pumpings. Thus, topological properties for acoustic pumping can be readily obtained in practice without requiring uniform pumping parameters across the space-modulated materials.

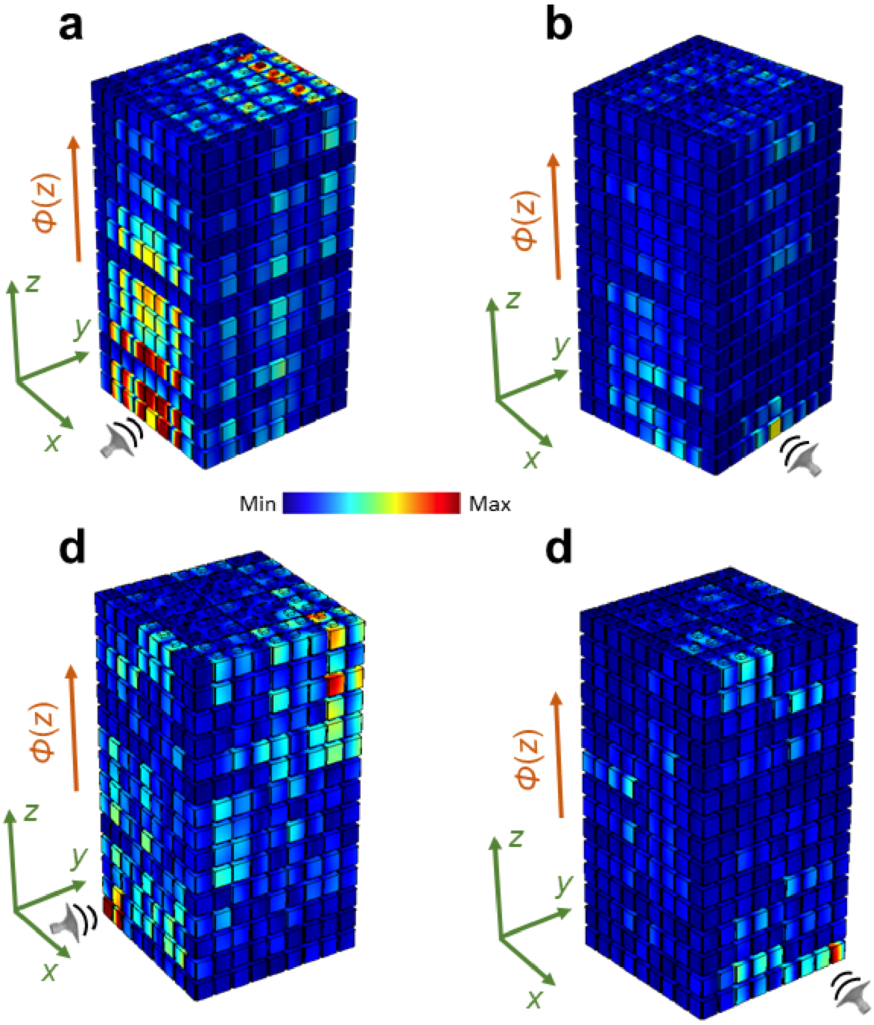

Supplementary Figure 10: **Acoustic path-dependent topological pumping along the diagonal orbit (the inset of Fig. 2d).** **a** Numerical observation of edge-to-edge topological pumping with the source placed on the left-bottom edge at $f = 7498$ Hz, while the pumping is completely absent if the source is moved to the right-bottom edge **b**. **c** Numerical observation of corner-to-corner topological pumping with the source placed on the left-bottom corner at $f = 6175$ Hz, while the pumping is completely absent if the source is moved to the right-bottom corner **d**.

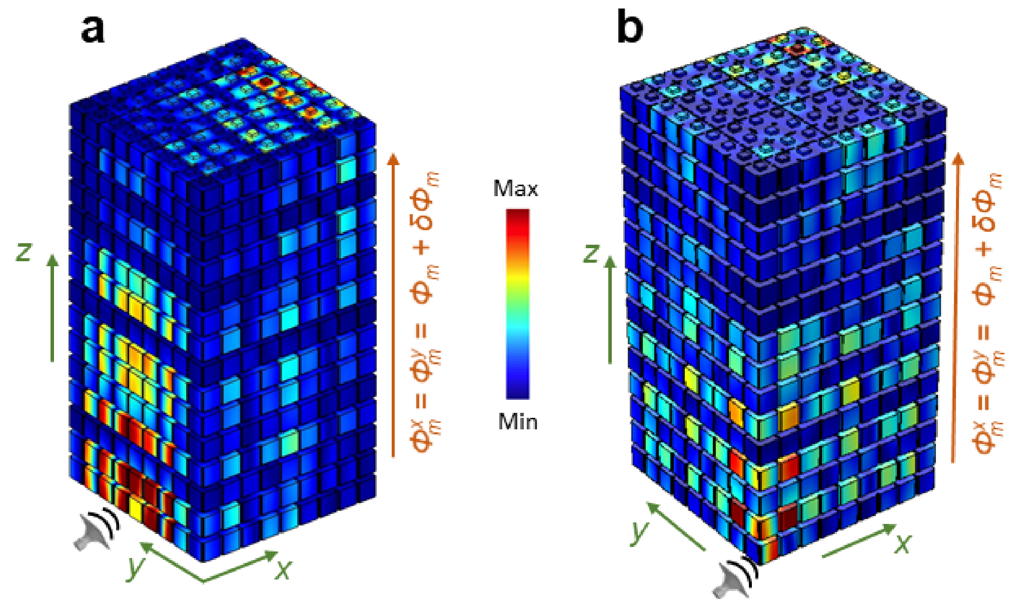

Supplementary Figure 11: **Robust sound transport to disorder in pumping parameters.** **a** Numerical observation of topological pumping of edge modes with random pumping parameters. Acoustic waves are injected at the left-bottom edge to be pumped to the right-top edge at frequency $f = 7498$ Hz. **b** Numerical observation of topological pumping of corner modes with random pumping parameters. Acoustic waves are injected at the bottom corner to be pumped to the diagonally opposite corner at the top layer with the frequency at $f = 6175$ Hz.

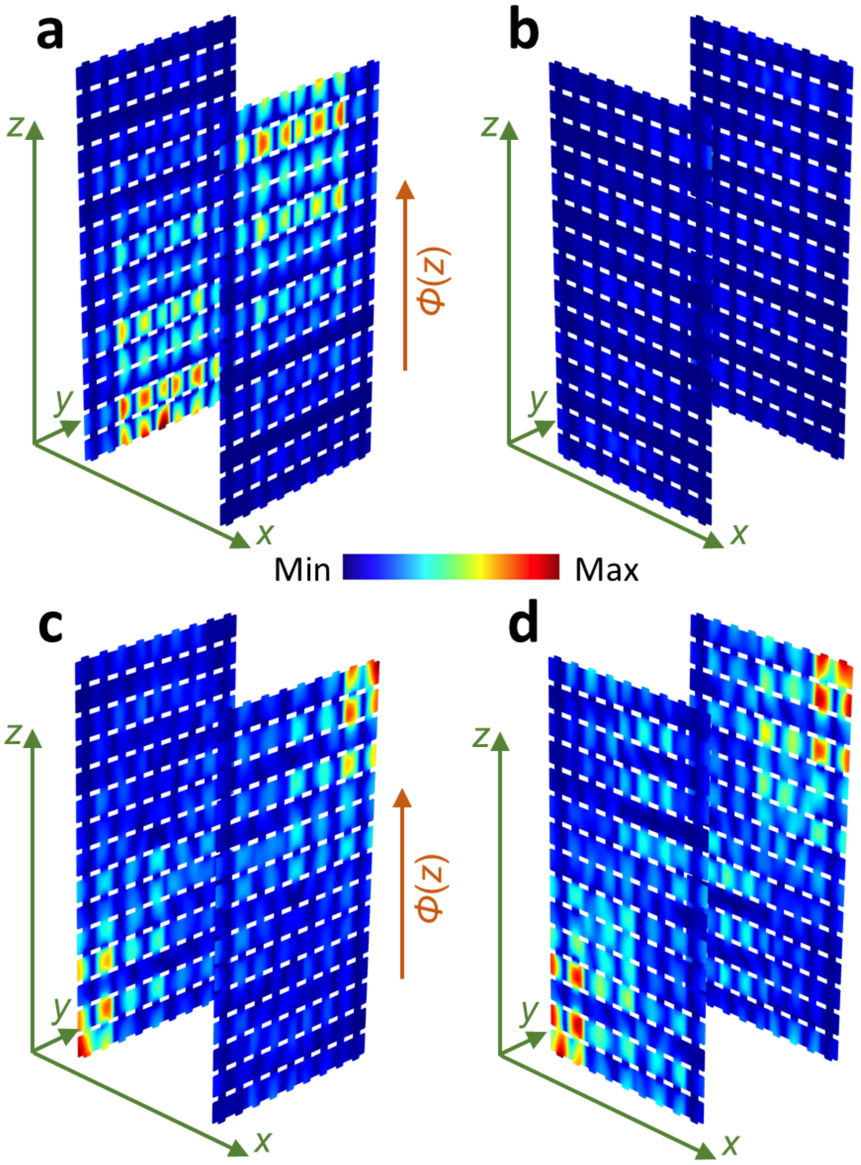

Supplementary Figure 12: **Topological pumping in the 3D channel-modulated acoustic crystal. a,b** Pressure distribution of edge-bulk-edge pumping along z-direction in boundaries. **c,d** Pressure distribution of corner-bulk-corner pumping along z direction in boundaries.

[1] Nakahara, M. *Geometry, Topology and Physics* (Oxford: Taylor & Francis, 2003), 2 edn.

[2] Zilberberg, O. *et al.* Photonic topological boundary pumping as a probe of 4D quantum Hall physics. *Nature* **553**, 59–62 (2018).

[3] Zhang, S.-C. & Hu, J. A four-dimensional generalization of the quantum Hall effect. *Science* **294**, 823–828 (2001).

[4] Chen, Z.-G., Zhu, W., Tan, Y., Wang, L. & Ma, G. Acoustic realization of a four-dimensional higher-order chern insulator and boundary-modes engineering. *Phys. Rev. X* **11**, 011016 (2021).
