# Supplementary material for: Creating synthetic spaces for higher-order topological sound transport
Source: Nat Commun. 2021 Aug 19;12:5028. doi: 10.1038/s41467-021-25305-z (PMC8377151; doi:10.1038/s41467-021-25305-z)
Supplement: Supplementary file 3 — Description of Additional Supplementary Files [file 41467_2021_25305_MOESM3_ESM.docx]

**Description of Additional Supplementary Files**

**Supplementary Movie 1.** Transient topological edge pumping in the 2D channel-modulated acoustic system. Numerical calculations of sound pressure field in response to excitation at the centre cavity of the left edge. The incident sound is a tone burst signal centred at ݂ = 4960 Hz.

**Supplementary Movie 2**. Transient topological edge pumping in the 3D channel-modulated acoustic system. Numerical calculations of sound pressure field in response to excitation at the centre cavity of the leftbottom edge. The incident sound is a tone burst signal centred at ݂ = 7498 Hz.

**Supplementary Movie 3.** Transient topological corner pumping in the 3D channel-modulated acoustic system. Numerical calculations of sound pressure field in response to excitation at the cavity of the left-bottom corner. The incident sound is a tone burst signal centred at ݂ = 6175 Hz
